# The dire side of autophagy in aging: Lessons from *C. elegans*

**DOI:** 10.1080/19420889.2017.1395120

**Published:** 2017-12-14

**Authors:** Rebeca Medina, Holger Richly

**Affiliations:** Laboratory of Molecular Epigenetics, Institute of Molecular Biology (IMB), Mainz, Germany

**Keywords:** aging, autophagy, ubiquitin proteasome system, neurodegeneration, *C. elegans*

## Abstract

Autophagy is an essential cellular process that eliminates cellular debris, dysfunctional organelles and misfolded proteins thereby maintaining cellular metabolism and homeostasis. Functional autophagy is generally thought to counteract aging and is crucial for the prolonged lifespan and health of an animal. Whereas this statement is true for young animals, we have recently shown that autophagy becomes dysfunctional in post-reproductive *C. elegans* contributing to aging and neurodegeneration. Downregulation of autophagy nucleation in post-reproductive nematodes causes a lifespan extension and preservation of neuronal integrity putting the generally accepted view of the role of autophagy in aging into question. Here we discuss specific cellular degradation programs in young and old animals and speculate on molecular mechanisms that might explain this unexpected role of autophagy in aged animals.

Macroautophagy (referred here to as autophagy) is a highly conserved bulk degradation program in which dysfunctional organelles and misfolded proteins are enclosed by double membrane structures to form autophagosomes. Upon fusion with lysosomes and subsequent exposure to hydrolytic enzymes the internalized content is degraded and recycled. Autophagy facilitates the constitutive turnover of dysfunctional cellular components, and allows the organism to adapt to cellular stress conditions such as nutrient deprivation or hypoxia. The failure of autophagy is generally believed to be one of the main reasons for the accumulation of cell damage and aging.^1,2^ Besides autophagy the cell exhibits a highly specific system, the ubiquitin proteasome system (UPS), which contributes to protein homeostasis and enables the tight control of cellular processes such diverse as gene regulation, apoptosis and DNA repair.^3,^^4^ The UPS plays an important role in preventing the formation of large protein aggregates, so called aggresomes, which cannot be degraded by the proteasome and rather constipate the proteasome and inhibit its catalytic activity.^5^ Thus, one important function of autophagy that complements the function of the UPS is the clearance of protein aggregates. Both degradation systems together are essential to maintain proteostasis and to prevent the aggregation of proteins, counteracting neurodegeneration and age-related diseases such as Alzheimer's, Parkinson's, and Chorea Huntington. We have recently conducted a study in the nematode *C. elegans* that has important implications for the current understanding of autophagy in the aged animal.^6^ Whereas in healthy, young animals both the UPS and autophagy contribute to proteostasis and clearance of cellular debris, in post-reproductive animals autophagy becomes dysfunctional affecting lifespan and healthspan of the nematode (Fig. 1). Our findings further indicate that the UPS is still working at full capacity in aged animals, but unlike autophagy, the UPS does not exhibit detrimental effects on the animal health.^6^ Of note, the maintenance of proteostasis via the UPS seems to come at a high price as a recent study on the quality control E3 ubiquitin ligase CHIP has demonstrated.^7^ However, our recent publication puts autophagy center stage in determining aging in post-reproductive animals. We demonstrate that organismal aging is accompanied by the accumulation of dysfunctional autophagosomes (Fig. 1) that are generated as a consequence of continuing autophagic vesicle nucleation but impaired autolysosome function primary in the neurons.^6^ This likely causes a blockage of the autophagic flux, which generates toxic effects that impact on lifespan and healthspan and which seems to be an immanent feature of the aging process in *C. elegans*. We discovered that post-reproductive inactivation of genes essential for autophagosome nucleation (Fig. 2), but not genes that control subsequent steps of the autophagic flux (vesicle elongation, maturation, and degradation), cause a significant lifespan increase and enhance neural integrity. Hence, by avoiding this detrimental autophagic deadlock in its early stages the toxic effects of dysfunctional autophagy are alleviated. On the flipside, downregulation of autophagy should create problems to dispose of damaged organelles, cellular debris and protein aggregates, which the UPS alone is unlikely to cope with.^8,9^ In addition, the selective clearance of organelles, as for example via mitophagy^10^ or via specific PINK-1 dependent vesicular trafficking pathways^11^ are unlikely to deal with the accumulation of dysfunctional organelles in *C. elegans* based on our findings.^6^ Thus, with regards to the underlying molecular mechanism for clearance of debris and protein aggregates we can only speculate at the current state of knowledge.

**Figure 1.**
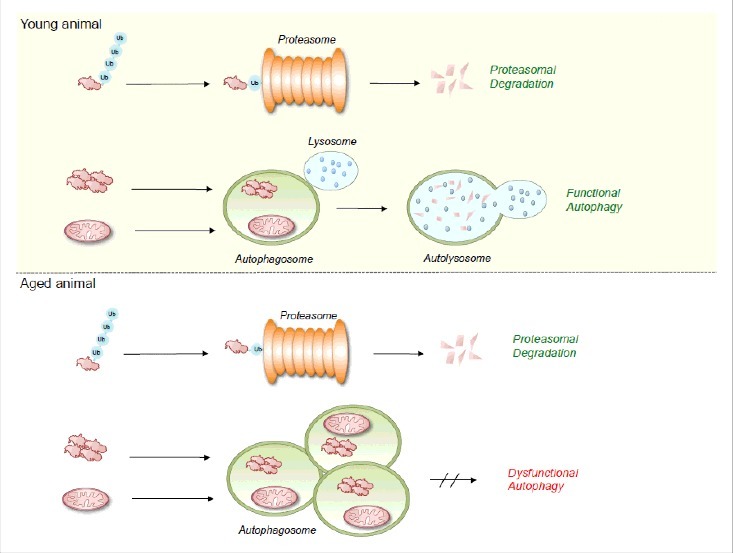
Clearance of cellular debris and maintenance of homeostasis in young and aged animals. In young animals ubiquitinated proteins are degraded by the 26S proteasome. Aggregated proteins, misfolded proteins and dysfunctional organelles are degraded by autophagy. In aged animals the UPS is still fully active whereas autophagy is dysfunctional causing an accumulation of autophagosomes and a block in the autophagic flux.

**Figure 2.**
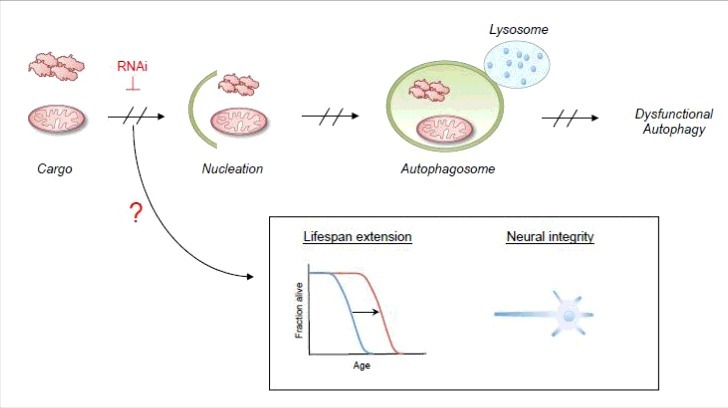
Downregulation of autophagic nucleation results in lifespan extension and neural integrity in *C.* *elegans*. Downregulation of vesicle nucleation by RNAi bypasses the autophagy pathway and results in lifespan extension and conservation of neurones by a yet unknown mechanism. Potential underlying mechanisms are discussed.

One important hallmark of neurodegenerative diseases is the accumulation of protein aggregates, which should typically be degraded via autophagy. In stark contrast, our study shows that neural integrity is preserved when shutting down autophagy in aged animals. Hence, the accumulation of dysfunctional organelles, misfolded proteins or the subsequent generation of aggresomes may either be tolerated in aged organisms to some degree or disposed of by a different clearance mechanism. A recent study in *C. elegans* has shown that protein aggregates and organelles existing in neurons can be internalized into vesicles, so-called exophers, which are transmitted into the sourrounding tissues.^12^ Importantly, this extrusion mechanism seems to specifically operate when autophagy or the UPS are inhibited.^12^ It could therefore represent a salvage pathway during age-related stress conditions, in which the autophagic flux and proteasomal degradation are dysfunctional. Downregulating autophagy in aged animals may have other beneficial physiological effects that contribute to health and longevity. Reducing the elevated age-related autophagosome formation might help to maintain the levels of cellular lipids that are essential for repair of cellular membrane structures, the lipid composition of plasma membranes and the distribution and function of lipid rafts and consequently membrane-associated signalling processes.^13^,^14^ In addition, studies with animals and with cell culture systems have shown that neurodegenerative diseases are caused by genetic mutations in the microtuble motor proteins, kinesin and dynein. Dysfunctional axonal transport seems to be the determinant in mediating pathology as for example observed in neurodegenerative diseases such as Alzheimer's and Parkinson's.^15^ Hence, accumulation of autophagosomes along the microtubule network might lead to a breakdown of the axonal retrograde and anterograde transport. The axonal transport is crucial for neuronal survival and impacting a wide range of cellular functions. Consequently, the aging-associated dysfunction of this neuronal transport system might contribute to neurodegeneration and ultimately cell death. Future research needs to aim at understanding the molecular mechanisms and the potential salvage pathways that are active when inhibiting autophagy in aged animals. A detailed, mechanistic insight might eventually open up new treatment strategies for neurodegenerative diseases.
